# Development of a Novel Co-Amorphous Curcumin and L-Arginine (1:2): Structural Characterization, Biological Activity and Pharmacokinetics

**DOI:** 10.3390/pharmaceutics17010011

**Published:** 2024-12-25

**Authors:** Jose Antonio Mancillas-Quiroz, Miriam del Carmen Carrasco-Portugal, Karina Mondragón-Vásquez, Juan Carlos Huerta-Cruz, Juan Rodríguez-Silverio, Leyanis Rodríguez-Vera, Juan Gerardo Reyes-García, Francisco Javier Flores-Murrieta, Jorge Guillermo Domínguez-Chávez, Héctor Isaac Rocha-González

**Affiliations:** 1 Escuela Superior de Medicina, Instituto Politécnico Nacional, Ciudad de México 11340, Mexico; jamq_1992@hotmail.com (J.A.M.-Q.); jrsilverio61@yahoo.com.mx (J.R.-S.); juangreyesgarcia@gmail.com (J.G.R.-G.); fjfloresmurrieta@yahoo.com.mx (F.J.F.-M.); 2 Instituto Nacional de Enfermedades Respiratorias, Ismael Cosío Villegas, Ciudad de México 14080, Mexico; miris22@hotmail.com (M.d.C.C.-P.); pharman007@hotmail.com (J.C.H.-C.); 3 Facultad de Bioanálisis Veracruz, Universidad Veracruzana, Veracruz 91700, Mexico; kmondragon@uv.mx; 4 CTI Clinical Trial & Consulting Clinical Research Center, Norwood, OH 45212, USA; leyanisrv80@gmail.com

**Keywords:** bioavailability, co-amorphous, curcumin, cytokines, inflammation, L-arginine, nociception, pharmacokinetics, solubility, stability

## Abstract

**Background:** Curcumin appears to be well tolerated and effective for managing chronic inflammatory pain, but its poor oral bioavailability has been a hurdle in its use as a therapeutic agent. The current study was performed to characterize a novel co-amorphous compound based on curcumin/L-arginine 1:2 (CAC12). **Methods**: Stability, solubility and structural characterization of the CAC12 were carried out by spectrometry techniques and in vitro assays, whereas the antinociceptive and anti-inflammatory effects were evaluated by CFA or carrageenan models. The mechanism of action was determined by cytokine quantification, and pharmacokinetic parameters were obtained through UPLC-MS/MS. The co-amorphous compound was prepared by fast solvent evaporation. Powder XRD, ^13^C-NMR, ATR-FTIR and TGA/DSC thermal analysis showed a 1:2 stoichiometry for the CAC12. **Results**: CAC12 was 1000 times more soluble than curcumin, and it was stable for 1 month at 40 °C and 75% relative humidity or for 60 min in physiological medium at pH 4.5–6.8. Co-amorphous curcumin/L-arginine, but not curcumin + L-arginine, decreased carrageenan- or CFA-induced inflammation and nociception by decreasing IL-1α, IL-1β, IL-6, TNF-α, MCP-1 and CXCL1 cytokines. The bioavailability of free plasmatic curcumin increased about 22.4 times when it was given as CAC12 relative to a phytosome formulation at the equivalent dose. **Conclusions**: Results suggest the possible use of CAC12 to treat inflammatory pain disorders in human beings.

## 1. Introduction

Pain is defined as “an unpleasant sensory and emotional experience associated with, or resembling that associated with actual or potential tissue damage” [1]. It is estimated that the worldwide prevalence of pain in adults over 25 years of age is around 27.5%, whereas in Mexico, the prevalence is about 22.2% [2]. Pain is a leading cause of disability worldwide and constitutes a major public health concern. The presence of inflammation is a common mechanism in acute and chronic pain and represents the hallmark of many painful conditions, such as joint inflammation, osteoarthritis and rheumatoid arthritis [3].

Non-steroidal anti-inflammatory drugs (NSAIDs) are the first-line treatment for inflammatory pain, incorporating approximately 60% of the over-the-counter pain therapy in the United States. However, the use of NSAIDs is limited since dose escalation or chronic use is associated with a greater risk of kidney disease, gastrointestinal damage, hepatotoxicity or coagulopathies [4]. At the gastrointestinal level, NSAIDs induce gastric ulcers in 10 to 30% of patients and increase the risk of lower gastrointestinal bleeding by between 1.9 and 18.4 times [5,6]. It has been calculated that about 107,000 people attend a hospital emergency department annually for NSAID-associated gastrointestinal adverse events in the United States, with a mortality rate of about 6% [7,8]. The above-mentioned figures highlight the need to develop effective analgesics with better safety profiles.

Curcumin is a hydrophobic polyphenol derived from the rhizome of the plant *Curcuma longa*, which is commonly used as a dietary supplement for its beneficial effects on health due to its antioxidant, neuroprotective, hypolipidemic, cardioprotective, hypoglycemic and anti-inflammatory properties, among others [9]. In clinical studies, curcumin shows an acceptable safety profile due to its ability to induce very low or no toxicity in subjects taking up to 8000 mg/day of this drug orally over a period of 3 months [10,11,12]. In fact, curcumin is classified as a substance Generally Recognized As Safe (GRAS) by the Food and Drug Administration [12].

Despite its safety profile and potential therapeutic uses, pharmacokinetic studies have demonstrated that oral bioavailability of curcumin is low due to its low solubility, poor intestinal absorption, extensive hepatic and intestinal metabolism and rapid clearance [11,13,14]. In relation to this, curcumin has been co-administered with adjuvants such as piperine to delay its metabolism [15,16]; consequently, innovative formulations based on emulsifiers such as carbohydrate complexes, polysorbates, phospholipid complexes and nanopreparations, among others, have been developed to increase the solubility and absorption of curcumin [16,17,18].

The formulation of co-amorphous drug delivery systems is a promising and emerging approach for improving the physicochemical properties of pharmaceuticals. In this regard, the formation of a co-amorphous solid of curcumin with a suitable co-former offers the potential to improve its solubility, bioavailability and/or stability [19]. L-arginine seems to be a good co-former, capable of improving the physicochemical properties of several drugs such as ibuprofen and indomethacin [20], naproxen [21], ciprofloxacin [22] or glibenclamide [23], among others [24,25,26].

Based on the aforementioned considerations, in the current study, we prepared and characterized a novel co-amorphous compound of curcumin/L-arginine (CAC12). Stability and solubility were measured by in vitro assays, structural characterization was unraveled by spectrometric methods, the anti-inflammatory and antinociceptive effects were evaluated through complete Freund adjuvant (CFA) or carrageenan animal models, the mechanism of action was elucidated by cytokine quantification, and bioavailability was evaluated through a pharmacokinetic clinical study in healthy subjects.

## 2. Materials and Methods

### 2.1. Synthesis of Co-Amorphous Curcumin/L-Arginine

The co-amorphous compound was prepared using the fast solvent evaporation technique. Briefly, 100 mg of a stoichiometric mixture of curcumin/L-arginine (1:2) was placed in a round flask and dissolved completely in 100 mL of methanol. Then, the solvent was evaporated in a rotary evaporator at 80 °C under vacuum until foam was formed in the flask. The resulting co-amorphous compound was scraped and placed in vials for further analysis. Further details of the synthesis and characterization of the co-amorphous compound can be found in Domínguez Chávez et al., 2019 [27].

### 2.2. Compound Sources

Complete Freund’s Adjuvant (CFA), carrageenan and diclofenac were purchased from Sigma-Aldrich (St. Louis, MI, USA), synthetic curcumin was acquired from Laurus Labs (Laurus Lab, Hyderabad, India), curcumin phytosome was obtained from iHerb Super Nutrition (Meriva^®^, Irvine, CA, USA), curcumin C3 complex^®^ was acquired from Sabinsa Corporation (Payson, UT, USA) and L-arginine was kindly provided by Laboratories Senosiain S.A. of C.V. (Guanajuato, Mexico). The capsules with turmeric extract phytosome (reference compound) for the pharmacokinetic study were purchased from Teva Pharmaceuticals Mexico S.A. de C.V. (Mericart^®^, CDMX, Mexico), whereas the capsules of CAC12 (test compound) were provided by Laboratories Senosiain S.A. de C.V. (CurQsen^®^, Guanajuato, Mexico).

For the preclinical studies, synthetic curcumin, L-arginine, diclofenac or CAC12 were dissolved in an isotonic saline solution with 10% polysorbate 80, and they were orally administered 1 h before CFA or carrageenan injection. CFA was administered at a concentration of 1 mg/mL and was previously dissolved in a mixture of paraffin oil (85%) and mannard monooleate (15%), whereas carrageenan was dissolved in an isotonic saline solution at 1%. The drugs were prepared fresh at the desired dose before administration.

### 2.3. Animals

Male Wistar rats weighing 140–200 g were used for the preclinic experiments. The animals were obtained from the vivarium of the Universidad Autonoma Metropolitana Xochimilco (CDMX, Mexico) and kept in our facilities with food (LabDiet 5010) and water ad libitum in a 12 h light/dark cycle and 23–25 °C until the day of the experiment. Every effort was made to minimize pain and suffering in animals, and the number of rats used was the minimum required to obtain a reliable statistical difference. Each rat was tested only once, and they were subjected to euthanasia in a carbon dioxide chamber at the end of the experiment.

### 2.4. Subjects

The pharmacokinetic study included 18 healthy subjects (9 males and 9 females) between 18 and 55 years of age. The exclusion criteria were hypersensitivity to curcumin or any other drug, overweight, obesity, alterations in vital signs or clinic parameters, ingestion of medications during the previous 7 days, hospitalization or blood donation in the last 3 months, consumption of tobacco, xanthines or roasted foods in the last 72 h, pregnancy, and consumption of alcohol or other substances of abuse. Informed consent was obtained from all subjects.

### 2.5. Powder XRD

The analysis was performed using a D2 Phaser diffractometer AXS (CuK*_α_*_1_ = 1.54184 Å) equipped with an SSD160 LynxEye detector (Bruker, Karlsruhe, Germany). The operating voltage and current were maintained at 300 kV and 10 mA, respectively. Scans were taken with a step size of 0.0303° and a counting rate of 0.300 s/step in a range of 2Ɵ from 5 to 60°. Samples were filled into a polymethylmethacrylate holder and gently compressed by a spatula until a compact sample with a smooth surface was obtained.

### 2.6. Solid-State ^13^C-NMR

The solid-state cross-polarization magic-angle-spinning ^13^C-NMR spectra for curcumin, L-arginine and CAC12 were recorded at room temperature using a 500 MHz Avance III HD spectrometer equipped with a cross-polarization magic angle spinning sequence pulse (Bruker, Karlsruhe, Germany). The experimental conditions were as follows: MAS spinning at 15 kHz, pulse delay of 10 s, contact time of 2 min and analysis time of 1 h 35 min.

### 2.7. FTIR-ATR Spectroscopy

Samples were analyzed by a diamond crystal attenuated total reflectance–Fourier transform infrared spectrometer Nicolet iS50 (Thermo Fisher Scientific, Madison, WI, USA). Spectra were collected using 64 scans with a resolution of 4 cm^−1^ over the range 400 to 4000 cm^−1^.

### 2.8. Thermal Analysis (TGA/DSC)

DSC thermal analysis was carried out using a Q2000 DSC analyzer equipped with a refrigerated cooling system 90 under a nitrogen gas flow of 50 mL/min (TA Instruments, Waters, Wilmington, DE, USA). The calibrations for temperature and enthalpy were performed using indium as a standard. For the experiment, about 2 mg of the co-amorphous compound was placed on aluminum pans (non-hermetically sealed) and heated at 20 °C/min from 25 °C to 400 °C. The glass transition temperature (Tg) and melting point (Tm) were determined using TA Universal Analysis 2000 software, version 4.7 (TA Instruments, Waters, DE, USA).

On the other hand, the TGA thermogram was obtained by a Q50 TGA analyzer (TA Instruments, Waters, DE, USA) under a nitrogen gas flow of 50 mL/min. For the CAC12 analysis, 2 mg of sample was loaded in a platinum pan and mounted on the equipment microbalance previously tared. The sample was heated at 20 °C/min in the temperature range of 30 °C to 400 °C. The weight loss percentage was calculated using TA Universal Analysis 2000 software, version 4.7 (TA Instruments, Waters, DE, USA).

### 2.9. Indicative Stability Tests

For the indicative stability tests, 30 mg of the CAC12 was placed in climate-simulating chambers under 40 °C and 0% relative humidity (Chamber RI-23-1060-ABA, Revco-Thermo Scientific, Asheville, NC, USA), 50 °C and 0% relative humidity (Chamber E-51, Riossa Company, Mexico City, Mexico) and 40 °C and 75% relative humidity (Chamber IP 20, Binder GmbH, Tuttlingen, Germany). After 1 month, the samples were removed from the incubators to be analyzed by powder XRD to detect possible phase changes. The tests were performed twice.

The stability of the CAC12 was also determined after 12 h in distilled water (pH = 7.0), phosphate buffer (pH = 6.8), acetate buffer (pH = 4.5) and HCl solution (pH = 1.2). About 80 mg of the sample was placed in a glass vial with two drops of the respective solution to moisten the co-amorphous compound, and then they were stirred at 37.5 °C on a stirring/heating plate. After 12 h, a small amount (approximately 20 mg) was taken out of the vial, dried at room temperature (24 °C), and analyzed by powder XRD and FTIR-ATR to detect diffraction peaks due to crystallization or changes in the IR spectra. All buffer solutions were prepared according to US Pharmacopeia methodologies. The experiments were carried out in duplicate.

### 2.10. Solubility

To compare the solubility of CAC12 against synthetic curcumin from Laurus in distilled water and phosphate buffer at pH 6.8, both drugs were supersaturated in 5 mL of medium (1.3 g of CAC12 and 0.3 g of synthetic curcumin, respectively), and the solutions were stirred at 37.5 °C on a stirring/heating plate. Then, 200 µL aliquots were taken for 1, 5, 10, 30 and 60 min. The curcumin concentrations were obtained at 420 nm using UV/Vis spectrophotometer S-3100 with a diode array (Scinco, Seoul, Republic of Korea). In addition, CAC12 solubility was also contrasted with other commercial curcumin formulations, including synthetic curcumin (Laurus Labs, India), curcumin C3 complex (Sabinsa, Bengaluru, India) and curcumin phytosome (Indena S.p.A., Milan, Italy) in phosphate buffer (pH 6.8) with 1% sodium lauryl sulfate. All concentrations were calculated from the absorbance and the Lambert–Beer equation using a curcumin calibration curve (ε_420_ = 14,294.9 L_*_mol^−1^_*_cm^−1^). The solubility tests were performed in triplicate.

### 2.11. CFA- and Carrageenan-Induced Inflammation

To evaluate the anti-inflammatory effect of CAC12, we used Complete Freund’s Adjuvant (CFA) and carrageenan as inflammatory agents. In the CFA-induced inflammation model [28,29], the rats were briefly anesthetized with isoflurane, via nose cone, titrated to the lowest dose necessary to achieve non-response to distal tail pinch. During the anesthesia period, the animals received a percutaneous injection of 100 µL of CFA (1 mg/mL) inside the capsule of the tibiotarsal joint of the right hind paw. On the other hand, in the carrageenan model [30], conscious rats were subcutaneously injected with 50 µL of carrageenan (1%) in the plantar region of the right hind paw.

In both models, the paw volume was measured by a digital plethysmometer 7150 (Ugo Basile, Varese, Italy). To measure volume, the injected paw was marked around 1 cm above the tibiotarsal joint with an indelible pen. The paw was immersed up to the mark every time. The volume change in the paw was measured before and 1, 2, 3, 4, 6, 8 and 24 h after the intra-articular injection of CFA, or before and 1, 2, 3, 4 and 6 h after the subcutaneous carrageenan injection.

### 2.12. Thermal Hyperalgesia

The antihyperalgesic effect of the co-amorphous compound was evaluated using the thermal hyperalgesia model described by Dirig et al. [31], with slight modifications. In this model, the rats were placed in acrylic observation chambers on a thin transparent glass plate maintained at a constant temperature of 30 °C. After a 30 min acclimatization period in the chambers, the animals received an intra-articular injection of 100 µL of CFA (1 mg/mL) or an intra-plantar injection of 50 µL of 1% carrageenan. Paw withdrawal latency to the thermal stimulus was measured before and 1, 2, 3, 4, 5 and 6 h after injection of CFA or carrageenan. To avoid tissue damage, a cutoff time of 20 s was used.

### 2.13. Quantification of Cytokines

To explore the possible mechanism of action of the CAC12, the levels of 23 cytokines (G-CSF, GM-CSF, GRO/KC, IFN-ɣ, IL-1α, IL-1β, IL-2, IL-4, IL-5, IL-6, IL-7, IL-10, IL-12, IL-13, IL-17A, IL-18, M-CSF, MCP-1, MIP-1α, MIP-3α, RANTES, TNF-α y, VEGF) were measured using the Bio-Plex^®^ Multiplex Immunoassay System (Biorad, Hercules, CA, USA) according to the manufacturer’s instructions. Briefly, the rats received an oral administration of vehicle (isotonic saline solution with 10% polysorbate 80) or 100 mg CAC12. One hour later, some animals received an intra-articular injection of 100 µL of vehicle (suspension of 85% paraffin oil and 15% mannard monooleate) or CFA (1 mg/mL) in the tibiotarsal joint, and others were injected intraplantally with 50 µL of vehicle (isotonic saline solution) or 1% carrageenan. Then, the rats were subjected to euthanasia in groups of 4 before and 1, 2, 4, 6 and 8 h after the injection of vehicle, CFA or carrageenan in the right hind paw. The right hind paw was removed 1 cm above the tibiotarsal joint, and the soft tissue of the paw was homogenized using a Vibra-Cell ultrasonic cell disruptor for 20 s (Sonics & Materials Inc., Newtown, CT, USA) in phosphate buffer (pH 7.5, containing 150 mM NaCl, 50 mM Tris-HCl, 5 mM EDTA, 0.1% Triton X100, 2 mg/mL aprotinin, 2 mg/mL leupeptin, 2 mg/mL pepstatin and 100 nM phenylmethanesulfonyl fluoride) at 1 mL of PBS for every 200 mg of tissue. The homogenized samples were centrifuged at 15,000× *g* for 5 min at 4 °C and the supernatants were stored at −70 °C until use.

For the quantification of cytokines, 50 µL of the solution with magnetic spheres containing antibodies against the 23 cytokines were mixed with 50 µL of blank solution, standard solution or sample in each well of the plate. Then, the plate was incubated at room temperature for 30 min under constant stirring. After that, the plate was washed three times using the Bio-Plex Pro Wash Station (Biorad, CA, USA). Subsequently, 25 µL of the solution containing the detection antibodies for the 23 cytokines was added to each well and the plate was again incubated at room temperature for 30 min under constant stirring. After incubation, the plate was washed three times, and 50 µL of streptavidin peroxidase solution was added to each well. Then, the plate was incubated for the third time at room temperature for 10 min, washed three times, stirred for 1 min and read in the Bio-Plex^®^ Multiplex Immunoassay System. Data were expressed as pg/mL.

### 2.14. Pharmacokinetic Study

A prospective, descriptive, longitudinal, single-blind, two-treatment, two-period, crossover, balanced, randomized, 7-day washout study design was implemented to compare the bioavailability of curcumin phytosome (reference compound, 150 mg) versus CAC12 (test compound, 150 mg) in 18 healthy subjects (9 males, 9 females).

In each period of the study, the health status of the subjects was verified through medical history, physical examination, electrocardiogram and laboratory tests; subsequently, they were fasted for 11 h and catheterized. After that, all subjects received two capsules of 500 mg Mericart^®^ or one capsule of 638 mg Curqsen^®^ alternately during the two periods. Each capsule of Mericart^®^ contained 500 mg of curcuma extract phytosome equivalent to 75 mg of curcumin, while each capsule of Curqsen^®^ was formulated with 292 mg of CAC12 (1:2) equivalent to 150 mg of curcumin. Subjects were instructed to report any symptoms they experienced, and the physicians conducted an interview aimed at detecting any adverse events during the study.

Blood samples were taken before drug administration and 0.5, 0.75, 1, 1.5, 1.75, 2, 2.5, 3, 4, 6, 8, 10, 12 and 24 h post-dose administration. Plasma was obtained by centrifugation of the blood samples at 4000 rpm for 10 min. To measure the plasmatic-free curcumin concentration and its metabolites, plasma samples were analyzed through a solid phase extraction method using Oasis HLB sorbent cartridges (Waters, Milford, MA, USA) and an Acquity ultra-performance liquid chromatograph (Waters, MA, USA) coupled to an API 5500 QTRAP mass/mass spectrometer (Sciex, Toronto, Canada) under the following chromatographic conditions: injection volume of 2 µL, mobile phase composed for a mixture (15:85 *v*/*v*) of a solution of 0.2% formic acid in 5 mM ammonium formate and acetonitrile, flow rate of 0.2 mL/min and temperature of 10 °C. The concentration of each sample was obtained using a calibration curve according to NOM-177-SSA1-2013.

### 2.15. Statistical Analysis

For the solubility experiments, time courses of curcumin concentration (mg/mL) versus time were constructed using the mean ± standard error of the mean (S.E.M.) of three tests. The statistical difference between CAC12 and curcumin phytosome, synthetic curcumin or curcumin C3 complex was obtained by two-way ANOVA followed by Tukey’s test.

In the preclinic studies, data were expressed as the average ± S.E.M. of 6 animals per experimental group. For the inflammation and hyperalgesia experiments, time courses of volume change (ΔV in µL) versus time (h) or paw withdrawal latency time (s) versus time (h) were generated, respectively. From the time courses, the area under the curve (AUC) was calculated using the trapezoidal rule as a measure of the duration and intensity of the effects observed with the different treatments. Then, AUC was employed to construct dose–response curves and to compare the different treatments. The statistical differences between the vehicle and increasing doses of curcumin, L-arginine or CAC12 were analyzed using one-way ANOVA followed by Dunnett’s test, whereas the statistical differences between the different treatments were obtained by one-way ANOVA followed by Tukey’s test.

For cytokine quantification, time courses of the cytokine concentrations (pg/mL) against time (h) were constructed using the average ± S.E.M. of 3–4 animals per experimental group. Then, the AUC of each time course was calculated, and the data were used to determine statistical differences between treatments with one-way ANOVA followed by Newman–Keuls´ test.

In the clinical study, the plasmatic concentrations of free curcumin (ng/mL) and its main metabolites (curcumin glucuronide or curcumin sulfate) were plotted against time (h) to obtain the pharmacokinetic profile and pharmacokinetic parameters (K_e_, t_1/2_, t_max_, C_max_, AUC_0–24_ and AUC_0-inf_) of each healthy subject. After that, the data were analyzed and presented as the mean ± S.E.M. of 18 healthy subjects per experimental arm. To compare statistical differences in C_max_, AUC_0–24_ and AUC_0-inf_ between the reference compound and the test compound, a paired Student’s *t*-test was used.

In all cases, the statistical difference was considered significant when the value of *p* was less than 0.05. In the solubility experiments and preclinic studies, statistical analyses were performed in GraphPad Prism, version 9.0. On the other hand, the pharmacokinetic parameters were calculated using Phoenix WinNonlin, version 8.1. All graphs were generated using GraphPad Prism, version 9.0.

## 3. Results

### 3.1. Structural Characterization of CAC12

#### 3.1.1. Powder X-ray Diffraction (XRD)

**Figure 1:**
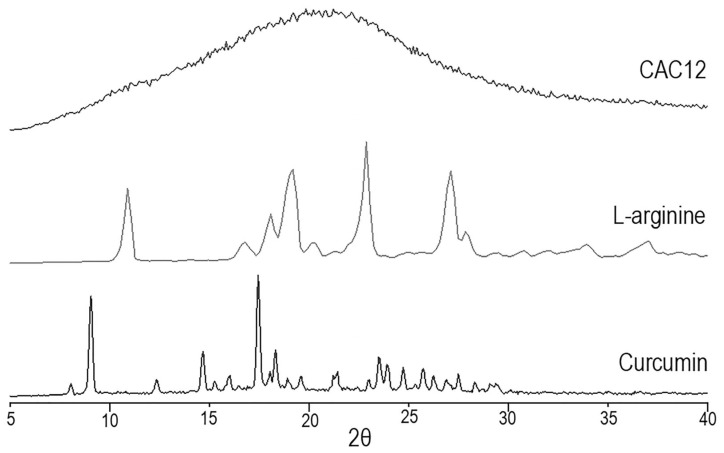
Powder X-ray diffractograms obtained for CAC12, L-arginine and curcumin.

CAC12 was obtained by vacuum-assisted rapid solvent evaporation. It was initially characterized by powder XRD and was compared with the diffractograms of the raw materials curcumin and L-arginine, as shown in Figure 1.

In the powder XRDs of curcumin and L-arginine, diffraction peaks are observed, indicating that the raw materials are crystalline, whereas the powder XRD of CAC12 shows the presence of a diffuse halo without diffraction peaks, which is an indication of the presence of an amorphous solid.

#### 3.1.2. Carbon-13 Nuclear Magnetic Resonance Spectroscopy (^13^C-NMR)

**Figure 2:**
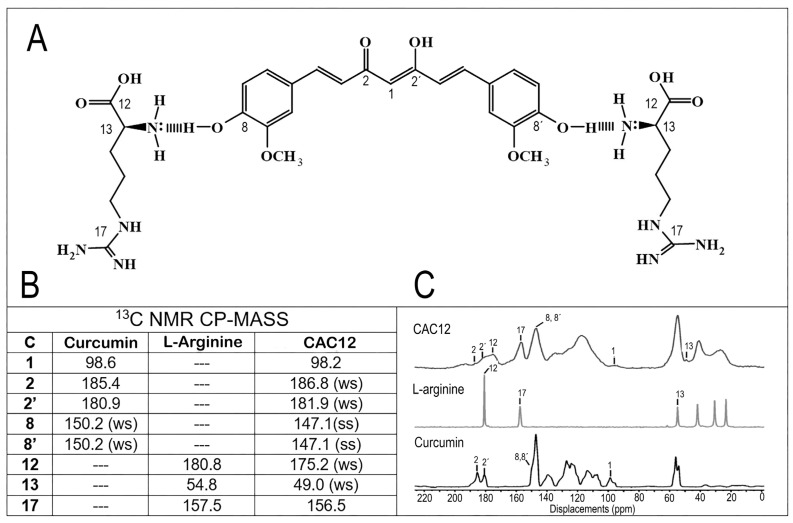
Solid-state carbon-13 nuclear magnetic resonance (^13^C-NMR) spectra of curcumin, L-arginine and CAC12. (**A**) Proposed chemical structure of CAC12. (**B**) The main displacements and (**C**) typical ^13^C-NMR spectra of CAC12, L-arginine and curcumin. Abbreviations: ws = wide signal, ss = small shoulder. As expected, the signals in the spectrum corresponding to the co-amorphous compound were observed to be broader and much less defined than those of the raw materials. In the CAC12 spectrum, we observed that the typical L-arginine signal at 54.8 ppm decreases to 49.0 ppm (Δδ = 5.8 ppm); likewise, the signal observed in the curcumin spectrum at 150.2 ppm, which is assigned to the hydroxyl-bonded aromatic carbons (C8 and C8′) shifts to 147.1 ppm (Δδ = 3.1 ppm) in the CAC12 spectrum.

To determine the interaction sites between curcumin and L-arginine in their amorphous state, ^13^C-NMR in the solid state was performed for CAC12, as well as for the raw materials curcumin and L-arginine. The proposed chemical structure of CAC12 and the resulting spectra are shown in Figure 2.

Another important shift in the spectrum of the CAC12 compound is that on the carbon of the carboxyl group of arginine, which shifts Δδ = 5.6 ppm to 175.2 ppm. It is fair to say that the signals at 98.6 ppm, 180.9 ppm and 185.4 ppm in the spectrum of curcumin corresponding to the carbonyl (C1) and enolic carbons (C2 and C2′), respectively, do not present significant displacement shifts (Δδ = 0.4, 1.4 and 1.0 ppm, respectively), Figure 2B,C.

#### 3.1.3. Attenuated Total Reflectance–Fourier Transform Infrared Spectroscopy (ATR-FTIR)

**Figure 3:**
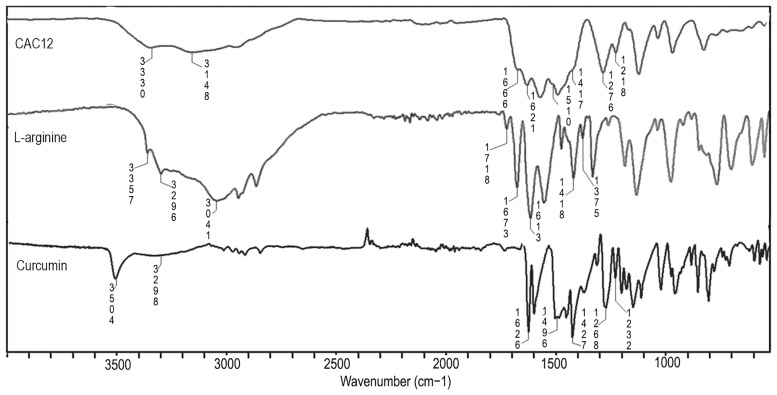
Attenuated total reflectance–Fourier transform infrared (ATR-FTIR) spectra for CAC12, L-arginine and curcumin.

To confirm the intermolecular interactions between curcumin and L-arginine in the co-amorphous state, we compared the ATR-FTIR spectra of curcumin, L-arginine and CAC12 (Figure 3, Supplementary Table S1). For curcumin, the most important bands are those associated with the phenolic hydroxyls (3504 and 1427 cm^−1^), carbonyl groups of the ketone (1626 and 1496 cm^−1^) and the enolic portion of curcumin (3298, 1268 and 1232 cm^−1^) [32,33,34,35]. For L-arginine, the main bands are those associated with the amino group (3357, 3041 and 1613 cm^−1^), the guanidinium group (3296 and 1673 cm^−1^) and the carboxylate group (1718, 1418 and 1375 cm^−1^) [36].

The CAC12 spectrum shows a broadening of the bands and, consequently, a loss in their definition, which is attributed to the amorphous solid, as determined by powder XRD and ^13^C-NMR in the solid state. The broadening of the bands makes it difficult to assign the vibrational bands and calculate their respective displacements. However, it is possible to observe the vibrational bands assigned to the phenolic hydroxyls of curcumin (3504 cm^−1^) and the amino group of L-arginine (3357 and 3296 cm^−1^) come together in a single broadband with a maximum at 3330 cm^−1^. Similarly, the bands at 1613 cm^−1^ and 1427 cm^−1^ assigned to the asymmetric deformation of the amino and the in-plane deformation of the phenolic C-OH bond of curcumin are shifted to 1621 cm^−1^ and 1417 cm^−1^, respectively, confirming the strong intermolecular interaction between the amino groups of L-arginine and the phenolic hydroxyls of curcumin, as was determined by solid-state ^13^C-NMR. In fact, the bands at 1621 cm^−1^, 1417 cm^−1^ and 1276 cm^−1^ are an indication of the formation of these intermolecular interactions in the CAC12 system. In addition, the bands assigned to the L-arginine carboxylate groups are slightly shifted at 1666 cm^−1^.

On the other hand, the bands assigned to the carbonyl and enol groups of curcumin did not undergo considerable shifts, which indicates that there are no other intermolecular interactions in CAC12.

### 3.2. Thermal Gravimetric Analysis/Differential Scanning Calorimetry (TGA/DSC)

**Figure 4:**
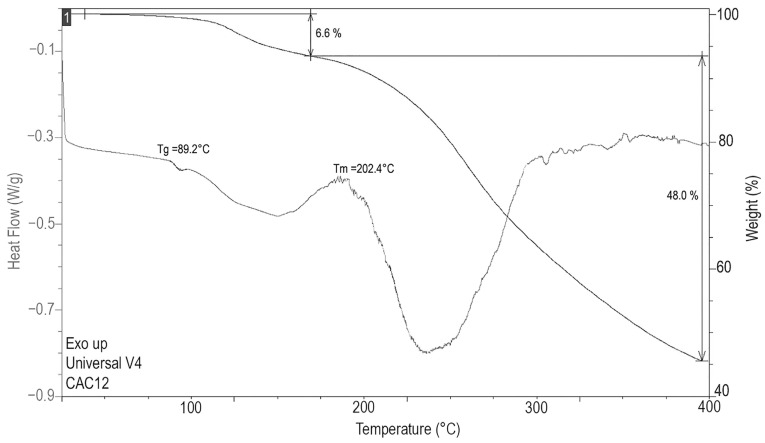
Differential scanning calorimetry (DSC) and thermal gravimetric analysis (TGA) of CAC12.

Thermal analysis of CAC12 was performed using TGA and DSC thermograms (Figure 4). In the DSC thermogram, the glass transition of CAC12 was detected at 89.2 °C, confirming its presence; a broad endothermic event was observed between approximately 110 °C and 200 °C, with a maximum at 150 °C, which is associated with the loss of 6.6% of the mass in the TGA thermogram and is attributed to the dehydration of 1.5 moles of water contained in the phases.

Subsequently, a new endothermic event is observed in the DSC thermogram at 202.4 °C that indicates the decomposition of the sample, which is associated with a weight loss of almost 50% of the sample in the TGA thermogram.

### 3.3. Indicative Stability Tests of CAC12

**Figure 5:**
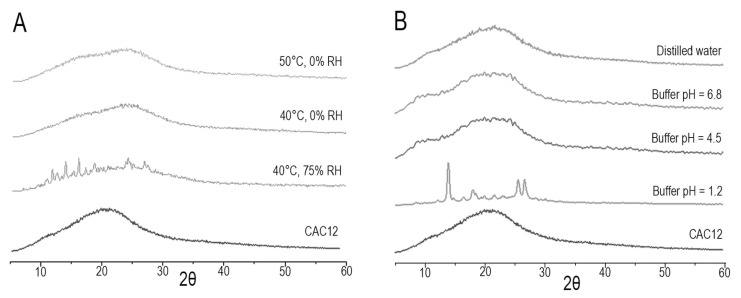
X-ray powder diffractograms acquired from CAC12 stability studies. (**A**) Powder X-ray diffractograms of CAC12 obtained after 1 month of storage under several temperature and relative humidity (RH) conditions. (**B**) Powder X-ray diffractograms of CAC12 obtained after 12 h in distilled water, phosphate buffer (pH = 6.8), acetate buffer (pH = 4.5) or HCl solution (pH = 1.2).

To evaluate CAC12’s stability, the compound was exposed to three environmental conditions of relative humidity (RH) and temperature (0% and 40 °C, 0% and 50 °C or 75% and 40 °C) for 1 month, and to a solution with different pH values (1.2, 4.5 and 6.8) for up to 12 h. Subsequently, the samples were characterized by powder XRD and compared to the initial powder XRD of CAC12 (Figure 5).

CAC12 remained unaltered at temperatures of 40 and 50 °C without RH, but when the co-amorphous compound was subjected to RH conditions (75%), diffraction peaks corresponding to the raw materials (curcumin and L-arginine) were observed in the powder XRD, indicating minor decomposition of the initial drug (Figure 5A). On the other hand, CAC12 did not show changes in the diffraction halo compared to its reference powder XRD when it was dissolved in distilled water, phosphate buffer (pH 6.8) or acetate buffer (pH 4.5) for 12 h; however, CAC12 completely dissociated after 12 h in hydrochloric acid (HCl, pH 1.2), as observed in Figure 5B.

### 3.4. Solubility of CAC12

The solubilities of CAC12 and synthetic curcumin in distilled water and phosphate buffer (pH 6.8) were tested. In the distilled water, the solubility of synthetic curcumin was 0.03 µg/mL and 0.43 µg/mL after 1 and 60 min, respectively, whereas the solubility of CAC12 was 344.51 µg/mL and 423.83 µg/mL after 1 and 60 min, respectively. In this regard, CAC12’s solubility in distilled water was 1000 to 10,000 times higher than that of synthetic curcumin throughout the time course. Similarly, in phosphate buffer (pH 6.8), the solubility of the co-amorphous compound was significantly higher than that of synthetic curcumin along the time course—the solubility of CAC12 was 220.41 and 354.65 µg/mL after 1 and 60 min, respectively, whereas the solubility of synthetic curcumin was only 1.36 and 3.75 µg/mL after 1 and 60 min, respectively. The time courses can be observed in Supplementary Figure S1.

In addition, CAC12’s solubility was compared with those of several trademarks of curcumin in distilled water with 1% sodium lauryl sulfate as the dissolution medium, according to the USP specifications for curcumin analysis. In the time course, CAC12’s solubility remained close to 6 mg/mL over 120 min; this concentration was about 30 times higher than those of curcumin phytosome, curcumin C3 complex and synthetic curcumin (Supplementary Figure S2).

### 3.5. Anti-Inflammatory Effects

**Figure 6:**
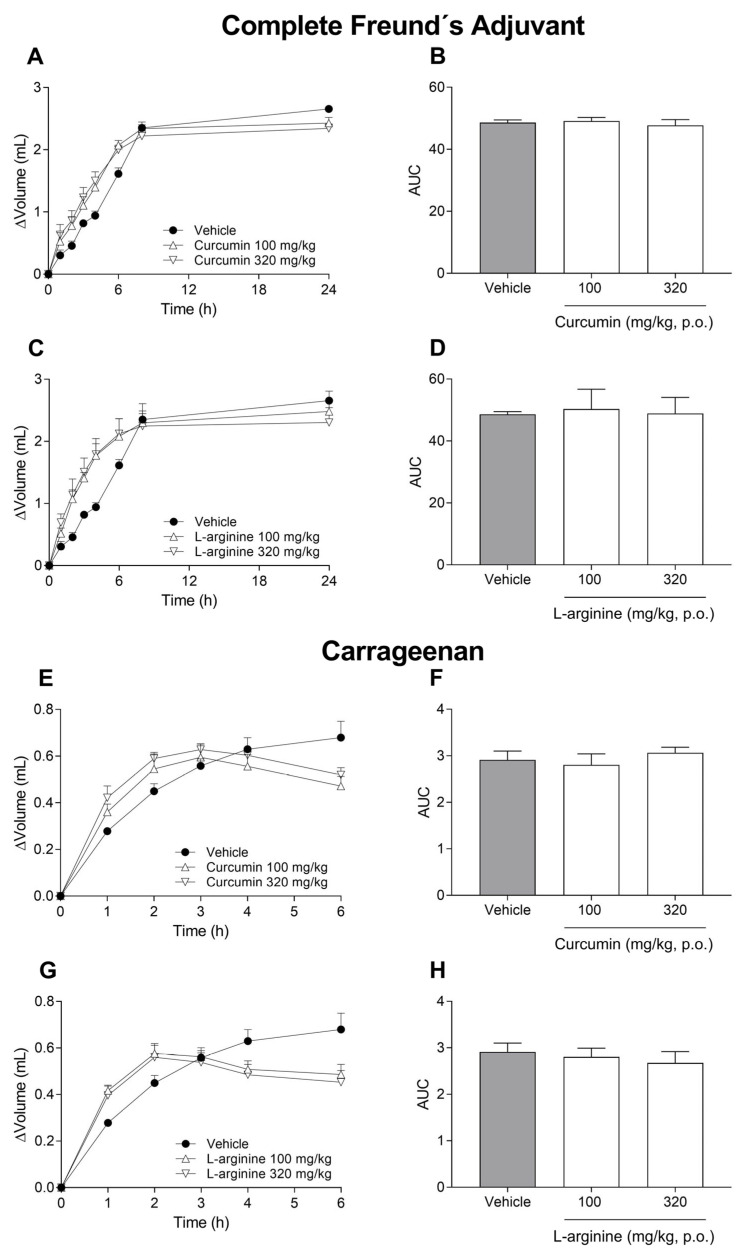
Lack of anti-inflammatory effect of oral curcumin and L-arginine in Freund’s complete adjuvant or carrageenan models. Time courses show the effects of increasing doses (100–320 mg/kg) of curcumin and L-arginine on inflammation induced by Freund’s complete adjuvant (**A**,**C**) or carrageenan (**E**,**G**). The bar graphs show a lack of anti-inflammatory effect of increasing doses of curcumin and L-arginine in Freund’s complete adjuvant (**B**,**D**) or carrageenan (**F**,**H**) models. Curcumin and L-arginine were orally administered at −1 h with respect to intra-articular injection of Freund’s complete adjuvant (100 µL, 0.1%) or subcutaneous administration of carrageenan (50 µL, 1%). Data are expressed as the mean ± S.E.M. of 6 animals per experimental group. There was no statistical difference based on one-way ANOVA. The area under the curve (AUC) was calculated from the time courses using the trapezoidal rule.

To evaluate the possible anti-inflammatory effects of curcumin, L-arginine and CAC12 on Freund’s complete adjuvant (CFA)- or carrageenan-induced inflammation, we measured rat paw volume using a plethysmometer. Oral administration of curcumin (100–320 mg/kg) or L-arginine (100–320 mg/kg) failed to reverse the inflammatory effects induced by CFA (Figure 6A–D) and carrageenan (Figure 6E–H).

**Figure 7:**
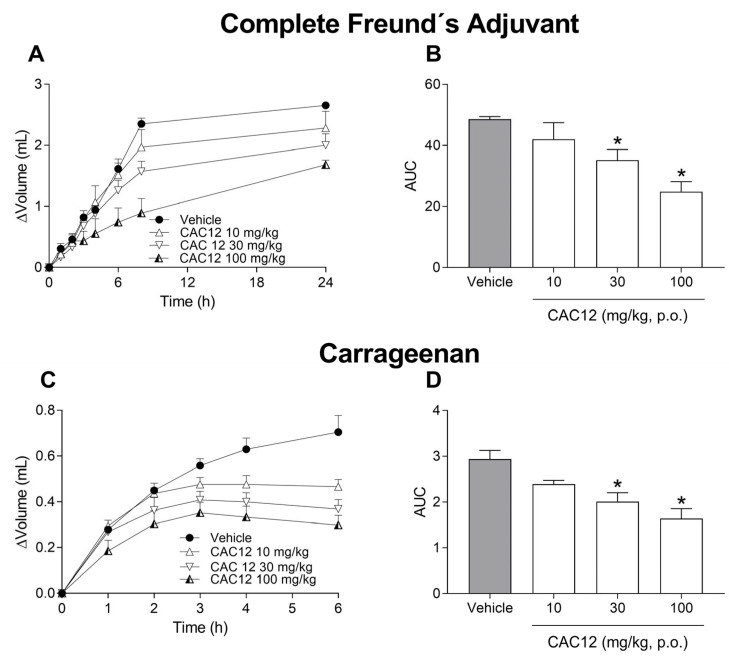
Anti-inflammatory effects of oral CAC12 (1:2) in Freund’s complete adjuvant or carrageenan models. Time courses show the effects of increasing doses (10–100 mg/kg) of CAC12 on inflammation induced by Freund’s complete adjuvant (**A**) or carrageenan (**C**). The bar graphs show the anti-inflammatory effect produced by increasing doses of CAC12 in Freund’s complete adjuvant (**B**) or carrageenan (**D**) models. Curcumin and L-arginine were orally administered at −1 h with respect to intra-articular injection of Freund’s complete adjuvant (100 µL, 0.1%) or subcutaneous administration of carrageenan (50 µL, 1%). Data are expressed as the mean ± S.E.M. of 6 animals per experimental group. * Statistically different from the vehicle based on one-way ANOVA followed by Dunnett’s test with *p* < 0.05. Statistical differences in time courses were omitted for the sake of clarity. The area under the curve (AUC) was calculated from the time courses using the trapezoidal rule.

On the contrary, oral administration of CAC12 (10–100 mg/kg) significantly reverted the inflammation induced by CFA (Figure 7A,B) and carrageenan (Figure 7C,D) in a dose-dependent manner. In both models, statistical differences with respect to the vehicle were reached at doses of 30 and 100 mg/kg (Figure 7B,D). Regarding the time courses, the 30 mg/kg CAC12 dose achieved a statistically significant anti-inflammatory effect compared to the vehicle group from the sixth hour onwards, whereas the 100 mg/kg CAC12 dose was statistically significant with respect to the vehicle from the sixth hour onwards in the CFA model (Figure 7A). Similarly, 10 mg/kg, 32 mg/kg and 100 mg/kg CAC12 doses had statistically significant anti-inflammatory effects with respect to the vehicle group from the fourth, third and second hours onwards, respectively (Figure 7B).

**Figure 8:**
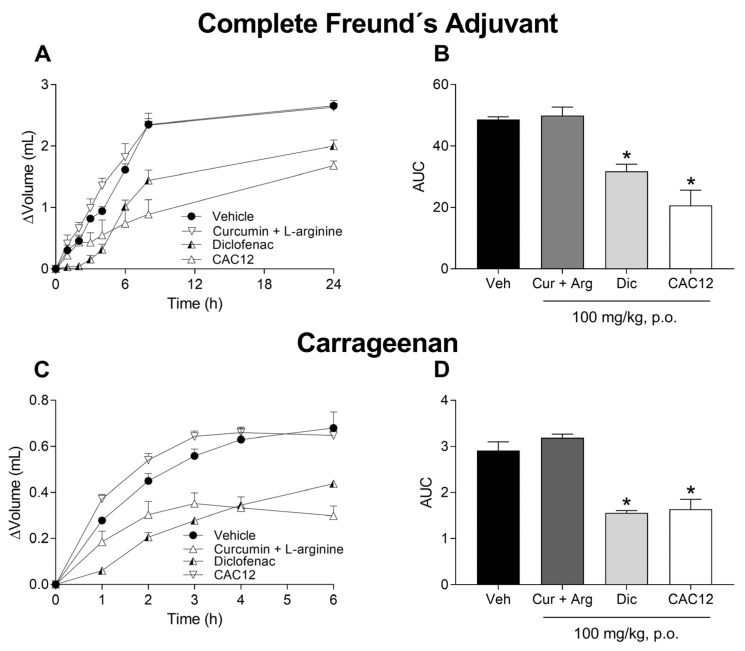
Effect of oral administration of 100 mg/kg of the crystalline mixture of curcumin (Cur, 51.4 mg) + L-arginine (Arg, 48.6 mg), diclofenac (Dic) and CAC12 (Co-amorph, 1:2) on inflammation induced by Freund’s complete adjuvant or carrageenan. The time courses show the anti-inflammatory effect produced by 100 mg/kg of diclofenac and CAC12 on the inflammation induced by Freund’s complete adjuvant (**A**) and carrageenan (**C**). The bar graphs indicate that 100 mg/kg of diclofenac and CAC12, but not the curcumin + L-arginine combination, have anti-inflammatory effects in Freund’s complete adjuvant (**B**) and carrageenan (**D**) models. Data are expressed as the mean ± S.E.M. of 6 animals per experimental group. * Statistically different from the vehicle group (Veh) based on one-way ANOVA followed by Tukey’s test with *p* < 0.05. Statistical differences in time courses were omitted for the sake of clarity. The area under the curve (AUC) was calculated from the time courses using the trapezoidal rule.

To determine if curcumin needs to be in an amorphous state to exert its anti-inflammatory effect, the animals received a single oral dose of 100 mg/kg CAC12 (1:2) or an equivalent dose of a crystalline mixture of curcumin (51.4 mg/kg) + L-arginine (48.6 mg/kg). CAC12, but not the physical mixture of curcumin + L-arginine, was able to reverse the inflammatory effect induced by CFA (Figure 8A,B) and carrageenan (Figure 8C,D). Furthermore, CAC12 induced an anti-inflammatory effect similar to that produced by diclofenac (positive control) in the CFA and carrageenan models.

### 3.6. Antihyperalgesic Effect

**Figure 9:**
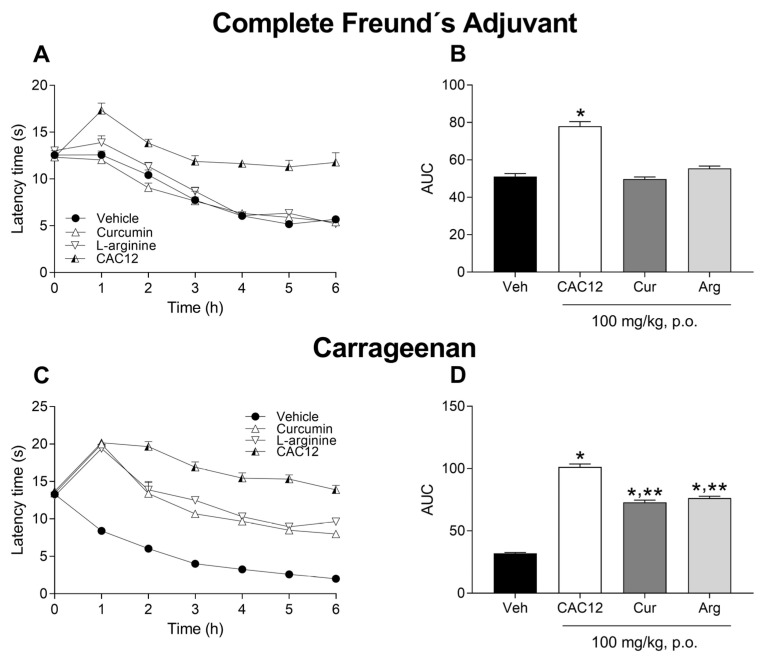
Effects of oral administration of 100 mg/kg of curcumin (Cur), L-arginine (Arg) and CAC12 (Co-amorph, 1:2) on nociception induced by Freund’s complete adjuvant or carrageenan. The time courses show the antinociceptive effect produced by 100 mg/kg of the CAC12 on the nociception induced by complete Freund’s adjuvant (**A**) and carrageenan (**C**). The bar graphs strongly suggest that 100 mg/kg of CAC12 exhibits an antinociceptive effect in Freund’s complete adjuvant (**B**) and carrageenan (**D**) models. Data are expressed as the mean ± S.E.M. of 6 animals per experimental group. * Statistically different with respect to the vehicle (Veh) group or ** CAC12 group based on one-way ANOVA followed by Tukey’s test with *p* < 0.05. Statistical differences in time courses were omitted for the sake of clarity. The area under the curve (AUC) was calculated from the time courses using the trapezoidal rule.

To compare the antihyperalgesic effects of single oral doses of CAC12 (100 mg/kg), curcumin (100 mg/kg) and L-arginine (100 mg/kg), we used CFA- and carrageenan-induced thermal hyperalgesia animal models. In the CFA-induced thermal hyperalgesia model, only the oral administration of CAC12, not curcumin or L-arginine, significantly increased the latency time with respect to the vehicle group (Figure 9A,B). On the other hand, in the carrageenan-induced thermal hyperalgesia model, curcumin, L-arginine and CAC12 showed antinociceptive effects that were statistically different from the vehicle group; however, the antihyperalgesic effect induced by CAC12 was statistically greater than curcumin or L-arginine (Figure 9C,D).

### 3.7. Mechanism of Action

To elucidate the possible mechanisms of action through which CAC12 produces its anti-inflammatory and antihyperalgesic effects, in the CFA- and carrageenan animal models, 23 cytokines involved in the inflammatory pain process were quantified after a single oral dose of 100 mg/kg of CAC12.

**Figure 10:**
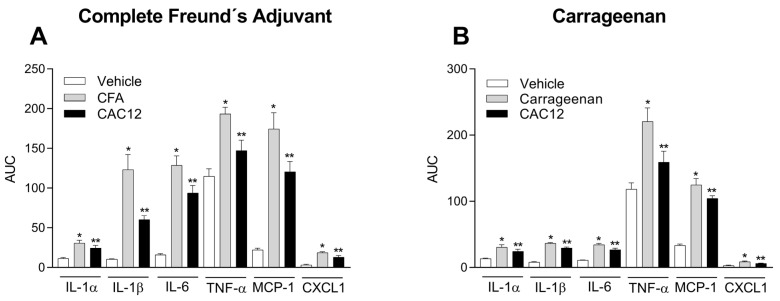
Effect of oral administration of CAC12 (1:2) on the levels of interleukin 1 alpha (IL-1α), interleukin 1 beta (IL-1β), interleukin 6 (IL-6), tumor necrosis factor-alpha (TNF-α), monocyte chemoattractant protein 1 (MCP-1) and CXC motif chemokine ligand 1 (CXCL1), induced by Freund’s complete adjuvant (**A**) or carrageenan (**B**). The bar graphs indicate that 100 mg/kg of CAC12 is able to prevent the increase in tissue concentrations of IL-1α, IL-1β, IL-6, TNF-α, MCP-1 and CXCL1 in Freund’s complete adjuvant (**A**) and carrageenan (**B**) models of inflammation for 8 h. Data are expressed as the mean ± S.E.M. of 3–4 animals per experimental group. * Statistically different from the vehicle group and ** statistically different from the carrageenan or CFA groups based on Newman–Keuls’ test with *p* < 0.05. The area under the curve (AUC) was calculated from time courses (from 0 to 8 h) using the trapezoidal rule.

The results indicated that the oral administration of CAC12 was able to decrease the elevation of interleukin 1 alpha (IL-1α), IL-1β, IL-6, tumor necrosis factor-alpha (TNF-α), monocyte chemoattractant protein 1 (MCP-1) and growth-related oncogene/keratinocyte-derived chemokine (GRO/KC), better known as the chemokine CXCL1, in both inflammation models (Figure 10). In addition, CAC12 was unable to modify the levels of IL-2, IL-4, IL-5, IL-7, IL-10, IL-12 (p70), IL-13, IL-17A, IL-18, granulocyte-colony stimulating factor (G-CSF), granulocyte-macrophage colony-stimulating factor (GM-CSF), macrophage colony-stimulating factor (M-CSF), macrophage inflammatory protein-1 alpha (MIP-1α), MIP-3α, regulated upon activation, normal T cell expressed and presumably secreted chemokine (RANTES), interferon-gamma (IFN-ɣ) or vascular endothelial growth factor (VEGF) in any of the models at the tested times. Time courses of cytokines that had a statistical difference are shown in Supplementary Figures S3 and S4.

### 3.8. Pharmacokinetics of Curcumin in the Co-Amorphous Compound

To establish whether curcumin in an amorphous state with L-arginine increases its bioavailability after a single oral dose, a pharmacokinetic study of the commercial product CurQsen^®^ comprising CAC12 was compared with a commercial product containing curcumin phytosome (reference compound, 150 mg) versus CAC12 (test compound, 150 mg) in 18 healthy subjects.

**Figure 11:**
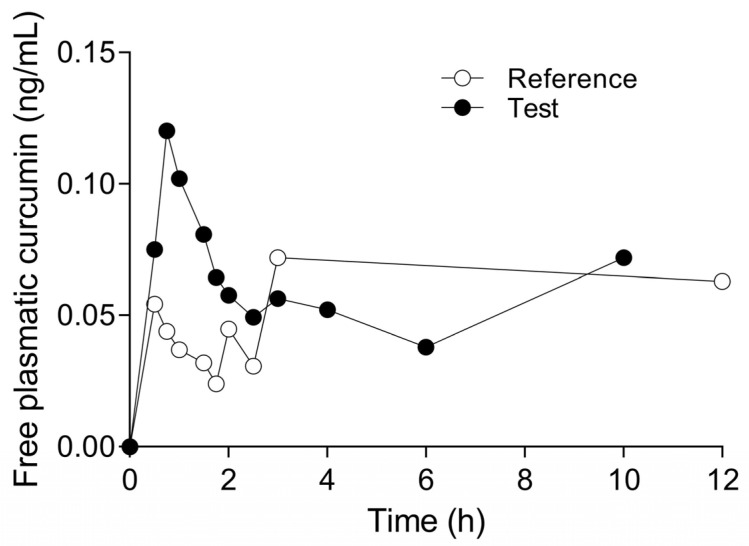
Pharmacokinetic profile of oral administration of the reference compound (curcumin phytosome, Mericart^®^) and the test compound (CAC12, CurQsen^®^). The data are expressed as the mean of the plasmatic-free curcumin of 18 healthy subjects.

The test compound showed a maximum concentration (C_max_) of free plasmatic curcumin that was about three times higher and statistically significant compared with the reference compound (0.12 ng/mL and 0.04 ng/mL, respectively). In addition, the half-life (t_1/2_) of the test compound was 8.2-fold longer than that of the reference compound (13.67 h^−1^ and 1.67 h^−1^, respectively), suggesting that curcumin remains available for a longer period of time to exert its pharmacological effect. Accordingly, the area under the curve at infinity (AUC_0-inf_) of free plasmatic curcumin in the test compound was 22.4 times greater and statistically significant compared with the reference compound (1.57 h·ng/mL and 0.07 h·ng/mL). The elimination rate constant (K_e_), t_1/2_ and AUC_0-inf_ could only be measured in a single subject in the reference compound group due to the low free plasmatic curcumin concentrations in this group. For this reason, the variability in these pharmacokinetic parameters could not be calculated (Figure 11, Table 1).

Regarding the main curcumin metabolites, the AUC_0-inf_ and the area under the curve at 24 h (AUC_0–24_) corresponding to the glucuronide curcumin in the test compound were significantly smaller than in the reference compound. In addition, the C_max_ of the test compound for glucuronide curcumin was lower than for the reference compound. Similarly, the pharmacokinetic parameters, C_max_, AUC_0-inf_ and AUC_0–24_, for sulfate curcumin in the test compound tended to be lower than in the reference compound (Table 1). There were no adverse events during the study.

## 4. Discussion

In the current study, we characterized a novel co-amorphous compound of curcumin with L-arginine as a co-former, which was synthesized via the fast solvent evaporation technique at a molar ratio of 1:2 for curcumin/L-arginine, respectively. Powder XRD acquired for L-arginine and curcumin showed typical diffraction peaks of crystalline compounds, whereas powder XRD of CAC12 showed a halo pattern, confirming the presence of a co-amorphous phase. Our diffractograms are in line with those previously reported for co-amorphous indomethacin/L-arginine, ibuprofen/L-arginine [20] and naproxen/L-arginine [21]. In this regard, the evidence indicates that L-arginine is a suitable co-former for physically stabilizing the amorphous form of analgesic and anti-inflammatory drugs.

It is well accepted that intermolecular interactions between a drug and a co-former are crucial in the formation of a co-amorphous system. To elucidate the molecular interaction sites between curcumin and L-arginine in our system, ^13^C-NMR and ATR-FTIR spectra of raw compounds and CAC12 were compared. In the ^13^C-NMR, the CAC12 spectrum exhibited displacements in the alpha carbon of L-arginine (54.8 ppm to 49.0 ppm) and the hydroxyl groups in the aromatic carbons of curcumin (150.2 ppm to 147.1 ppm), suggesting that the amino group of each L-arginine interacts with one phenol of curcumin. In a similar way, the carboxyl group of arginine also seems to establish intermolecular interactions since this group showed a displacement of 5.6 ppm on the CAC12 spectra. Conversely, the carbonyl and enolic carbons of curcumin did not show important displacements, indicating that the keto-enol part of curcumin does not participate in the formation of the co-amorphous system [37]. In addition, the peaks in the CAC12 spectrum were broader and less defined compared to the raw materials, which is attributed to the high degree of molecular disorder associated with the amorphous form.

Regarding the ATR-FTIR, curcumin and L-arginine spectra were in good accordance with previous publications [32,33,34,35,36]. In addition, the CAC12 spectrum showed overlapping bands due to their broadening, as expected in the co-amorphous state. The main changes detected in the CAC12 spectrum occur in the O-H and N-H vibration zones where the phenol stretching band of curcumin (3504 cm^−1^), amine asymmetric stretching of L-arginine (3357 cm^−1^) and amine symmetric stretching of L-arginine (3041 cm^−1^) peaks disappear or attenuate to form a wideband (3300 cm^−1^), implying that the phenolic hydroxyls of curcumin establish an important interaction with the amino groups of L-arginine. Similarly, there is a loss of definition and intensity in the bands observed between 1400 and 1600 cm^−1^, which is indicative of the absence of carboxylate ions in L-arginine salt and encourages us to point out that there is no proton transfer between L-arginine and curcumin since they are in the co-amorphous phase. Moreover, in this zone, there are slight shifts in the bands assigned to the asymmetric deformation of L-arginine and the in-plane deformation of curcumin phenols, reinforcing the argument that the amine of L-arginine is interacting with the curcumin phenols to form the co-amorphous system.

The DSC/TGA thermogram analysis is also in line with the observations found in the ^13^C-NMR and ATR-FTIR spectra since only a glass transition temperature was observed at 89.2 °C, which is a characteristic of homogeneous solids but not of physical mixtures. In addition, the CAC12 system seems to be hydrated because the thermogram showed an endothermic event (around 100 °C) that corresponded to a 6.6% weight loss (1.5 moles of water). After dehydration, CAC12 begins crystallizing at 190 °C and decomposing at 202.4 °C. In summary, the spectroscopic and thermal analyses indicate that the amino groups of L-arginine form hydrogen bonds with the hydroxyl groups of curcumin in the amorphous state.

The preparation of co-amorphous drug systems aims to improve the aqueous solubility and physical stability of drugs [37]. In our study, the indicative stability studies strongly suggested that CAC12 was stable between 40 °C and 50 °C under dry conditions for at least 1 month, but a small decomposition was observed when the co-amorphous drug system was stored at 50 °C and 75% RH. Typically, hygroscopicity is a major limitation of co-amorphous drug systems since the water acts as a plasticizer that increases the molecular mobility of the co-amorphous state, favoring the crystallization of the individual components. Thus, these data confirm that CAC12 is sufficiently stable at temperatures below the glass transition temperature (<90 °C) under dry conditions and also indicate that CAC12 should be formulated into capsules or coated tablets and packaged in blisters to avoid humidity and improve its long-term stability.

CAC12 also remained stable at pH 4.5–6.8, demonstrating that this system is stable at intestinal pH, a requirement for absorption to take place. On the contrary, CAC12 was dissociated immediately upon contact with HCl at pH 1.2, causing the precipitation of curcumin as a crystalline solid. The results show that the amine group of L-arginine is protonated in an acidic medium, leading to the breakdown of the interaction with curcumin and the precipitation of CAC12.

The main disadvantage of curcumin is its poor solubility in water (0.6 µg/mL) [38], which translates into low bioavailability and limited efficacy. In the current study, the CAC12 not only reached a solubility of up to 6000 mg/L in distilled water with 1% sodium lauryl sulphate, it was also 30 times greater than other trademark curcumin formulations. In this regard, the solubility of curcumin in our co-amorphous system indicates that the interaction between curcumin and L-arginine in solution is stable for at least 60 min, which is relevant because of the insolubility of curcumin in water and its short half-life in solution (around 10 min in phosphate buffer at pH 7.2) [38,39]. Thus, the current CAC12 system prevents the precipitation of curcumin and allows it to be in solution over a longer period of time. Furthermore, the data confirm that our CAC12 formulation has a greater solubility than synthetic curcumin or curcumin formulations developed using turmeric extracts (curcumin C3 complex) or emulsifiers (curcumin phytosome) [18].

Consistent with the solubility results, CAC12 increases the bioavailability of free plasmatic curcumin concentration in relation to a curcumin phytosome formulation in healthy subjects. Consequently, curcumin metabolites were detected in lower concentrations in the CAC12 group. Also, in the curcumin phytosome group, the free plasmatic curcumin concentration was undetectable in 15 of 18 subjects after 3 h, showing its low bioavailability. In fact, computation of standard deviations for the K_e_, t_1/2_ and AUC_0-inf_ parameters in this group was impossible due to the limited data obtained. Based on the low bioavailability of curcumin in plasma, the authors were unable to quantify free curcumin after a single dose of up to 12 g of curcumin C3 complex in a pharmacokinetic clinical study, but they detected the main curcumin conjugates over 24 h, indicating that curcumin is rapidly metabolized into curcumin sulphate and curcumin glucuronide [40]. Unfortunately, curcumin conjugates are ineffective due to their large size [18]. On the contrary, our results suggest that the pharmacokinetic profile of curcumin in the amorphous state is improved because it is absorbed in higher quantities, is less metabolized, and remains bioavailable in the plasma for a longer time with respect to the formulation based on phytosomes. CAC12 capsules contain black pepper powder extract, among other excipients. Black pepper powder can help to enhance the effect of curcumin since it inhibits its glucuronidation; however, it has been shown that although it can increase curcumin absorption, it is not able to maintain active curcumin levels in the blood. It is also necessary to administer at least 20 mg of black pepper powder extract to observe this effect, and in this case, as it is an excipient, it is not found in such quantities [41]. Thus, we suggest that CAC12 solubility is the main factor responsible for the higher absorption observed with our formulation.

Preclinic and clinical evidence shows that curcumin is a suitable candidate for treating inflammatory and pain disorders [42,43,44,45,46]; however, its potential efficacy has been questioned due to its low oral bioavailability [16,47]. Here, curcumin in a co-amorphous state, but not free curcumin, was able to decrease CFA- and carrageenan-induced inflammation and hyperalgesia in rats. Interestingly, the co-administration of a physical mixture of curcumin + L-arginine also failed to prevent CFA- or carrageenan-induced inflammation, demonstrating that curcumin needs to be administered in its co-amorphous state to exert its effect. In addition, the interaction between curcumin and L-arginine appears to be pharmacokinetic rather than pharmacodynamic since oral administration of L-arginine was ineffective in both inflammation models and CFA-induced hyperalgesia. Notwithstanding, pharmacodynamic interaction in carrageenan-induced hyperalgesia cannot be completely discarded.

In our study, the anti-inflammatory effect of curcumin in the amorphous state was similar to that of diclofenac in both animal models. Diclofenac is an NSAID that is widely utilized in clinics to treat and manage acute and chronic pain associated with inflammatory conditions; however, as with other NSAIDs, it is linked to serious dose-dependent gastrointestinal, cardiovascular and renal adverse effects [48]. In this regard, curcumin was safer than diclofenac in a comparative study [49], and it has been suggested that curcumin could be a good therapeutic alternative for NSAIDs since several clinical studies have demonstrated that curcumin is well tolerated from 1 to 8 g/day for 3–8 months, with mild diarrhea and nausea as the most reported adverse events [14,50,51]. In our pharmacokinetic study, no adverse effects related to curcumin occurred.

Previous studies have mentioned that the effect of curcumin on pain and inflammation is mediated by the regulation of pro-inflammatory and oxidative stress mediators; the modulation of enzymes (COX-2, CaMKIIα), ionic channels (TRP) and receptors (mGlu2, opioid); as well as the inhibition of JAK2/STAT3, JNK/MAPK and ERK/CREB signaling pathways, among others [12,42]. In the current study, CAC12 prevented the increase in IL-1α, IL-1β, IL-6, TNF-α, MCP-1 and CXCL1 induced by CFA or carrageenan. Data are consistent with the anti-inflammatory and antihyperalgesic effects observed in both animal models.

MCP-1 is a chemokine responsible for recruiting monocytes, neutrophils and lymphocytes, as well as inducing chemotaxis through the activation of G protein-coupled receptors. In addition, it is one of the key chemokines in the migration and infiltration of macrophages to the site of inflammation [52]. CXCL1 protein is a chemokine responsible for the recruitment of neutrophils in the early stage of the inflammatory process, and it amplifies the activation of the inflammasome in the carrageenan model in mice [53,54]. IL-1β, IL-6 and TNF-α are also cytokines that play an important role in acute and chronic inflammation; thus, IL-1α and IL-1β are released mainly from macrophages and neutrophils, and among other effects, they activate mast cells that release histamine, which, in turn, favors vasodilation and permeability of capillaries for the infiltration of more cells of the immune system. IL-6 is released by a wide variety of cells, such as mononuclear phagocytes, T cells and fibroblasts. Its main effects on the inflammatory process have to do with the maturation of B cells and the activation and differentiation of T cells. Finally, TNF-α is a product of macrophages, fibroblasts, mast cells and some killer T cells. Its main functions involve local activation of the vascular endothelium. It also favors vasodilation and increased vascular permeability, facilitates the activation of T and B lymphocytes and increases platelet activation and adhesion. Likewise, it is known that TNF-α is one of the cytokines that perpetuates the inflammatory response, for example, in rheumatoid arthritis, by activating the synthesis of other cytokines such as IL-6 [55,56].

All this evidence suggests that CAC12 has therapeutic potential for the treatment of pain associated with inflammatory disorders. However, further clinical studies are required to demonstrate its efficacy and safety in specific pathologies such as rheumatoid arthritis.

## 5. Conclusions

CAC12, prepared by the rapid solvent evaporation technique at a stoichiometric ratio of 1:2, was very stable under common storage conditions of temperature and RH, as well as in solutions with pH values between 4.5 and 6.8. In addition, in vitro studies indicated that the solubility of curcumin in the co-amorphous compound is around 30 times higher than any other formulation on the market. Consistently, oral administration of CAC12, but not oral co-administration of a physical mixture of curcumin + L-arginine, decreased CFA- and carrageenan-induced antihyperalgesic and anti-inflammatory effects in rats. Moreover, the bioavailability of curcumin in healthy subjects was significantly greater in CAC12 than in a commercial phytosome extract of curcumin.

Together, these data indicate that the CAC12 formulation improves the stability, solubility and bioavailability of curcumin, improving its ability to exert its anti-inflammatory and antihyperalgesic effects through inhibition of pro-inflammatory cytokines. The results indicate that the CAC12 formulation could be a good therapeutic alternative for treating inflammatory pain disorders in humans.

## 6. Patents

The patents associated with this work are listed below:

- World patent: WO/2021/044231. 2021 March 11. Spanish
- Mexican Patent: MX/a/2019/010692. 2019 September 6. Spanish.

## Figures and Tables

**Table 1:** Pharmacokinetic parameters of free curcumin, curcumin glucuronide and curcumin sulfate calculated after an oral administration of the reference compound (curcumin extract phytosome, Mericart^®^) or the test compound (CAC12, 1:2). The data are expressed as the mean ± S.E.M. of 18 healthy subjects.

Pharmacokinetics Parameters						
**Free curcumin**						
**Drug**	**k_e_** **(h^−1^)**	**t_1/2_** **(h)**	**t_max_** **(h)**	**C_max_** **(ng/mL)**	**AUC_0–24_** **(h·ng/mL)**	**AUC_0-inf_** **(h·ng/mL)**
Reference	0.42 ± ND	1.67 ± ND	1.04 ± 0.24	0.04 ± 0.01	0.10 ± 0.05	0.07 ± ND
Test	0.94 ± 0.21	13.67 ± 11.45	1.40 ± 0.21	0.12 ± 0.02 *	0.22 ± 0.05 *	1.57 ± 1.23
**Curcumin glucuronide**						
Reference	0.10 ± 0.02	56.63 ± 47.25	1.93 ± 0.35	16.72 ± 2.21	62.72 ± 6.61	212.20 ± 108.38
Test	0.42 ± 0.08	4.53 ± 1.99	1.61 ± 0.22	16.10 ± 2.31	45.07 ± 6.99 *	68.58 ± 15.03 *
**Curcumin sulfate**						
Reference	0.33 ± 0.17	15.94 ± 6.77	2.61 ± 0.64	0.99 ± 0.19	3.31 ± 0.51	12.30 ± 5.23
Test	0.30 ± 0.14	20.77 ± 14.26	2.79 ± 0.52	0.83 ± 0.14	3.48 ± 0.46	8.20 ± 2.63

* Statistically different from the reference compound by paired Student test, with a *p* < 0.05.

## Data Availability

The original contributions presented in the study are included in the article/Supplementary Materials; further inquiries can be directed to the corresponding authors.

## Supplementary Materials

The following supporting information can be downloaded at: https://www.mdpi.com/article/10.3390/pharmaceutics17010011/s1, Figure S1: Comparative solubility of curcumin as phytosome or CAC12 in distilled water and phosphate buffer (pH = 6.8) at 37.5 °C, Figure S2: Comparative solubility of curcumin as CAC12 (1:2), curcumin phytosome, curcumin C3 complex or synthetic curcumin in distilled water with 1% sodium lauryl sulphate, Figure S3: Time courses showing the effect of oral administration of 100 mg/kg of CAC12 (1:2) on the levels of interleukin 1 alpha (IL-1α), interleukin 1 beta (IL-1β), interleukin 6 (IL-6), tumor necrosis factor-alpha (TNF-α), monocyte chemoattractant protein 1 (MCP-1) and CXC motif chemokine ligand 1 (CXCL1) induced by complete Freund’s adjuvant (CFA), Figure S4: Time courses showing the effect of oral administration of 100 mg/kg of CAC12 (1:2) on the levels of interleukin 1 alpha (IL-1α), interleukin 1 beta (IL-1β), interleukin 6 (IL-6), tumor necrosis factor-alpha (TNF-α), monocyte chemoattractant protein 1 (MCP-1) and CXC motif chemokine ligand 1 (CXCL1) induced by complete carrageenan, Table S1: Summary of the main vibrational band assignments of curcumin, L-arginine and CAC12 by attenuated total reflectance–Fourier transform infrared.
