# Supplementary material for: Development of a Novel Co-Amorphous Curcumin and L-Arginine (1:2): Structural Characterization, Biological Activity and Pharmacokinetics
Source: Pharmaceutics. 2024 Dec 25;17(1):11. doi: 10.3390/pharmaceutics17010011 (PMC11768591; doi:10.3390/pharmaceutics17010011)
Supplement: Supplementary file 1 [file pharmaceutics-17-00011-s001.zip › pharmaceutics-3331444-supplementary.pdf]

Supplementary Figures

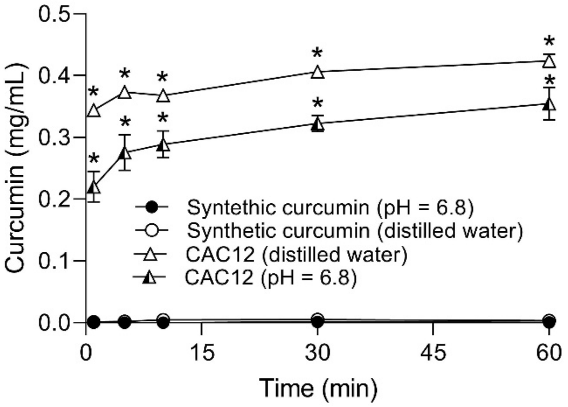

Figure S1. Comparative solubility between synthetic curcumin and CAC12 in distilled water and phosphate buffer (pH = 6.8) at 37.5 ºC. Data are expressed as the mean ± S.E.M. of 3 samples per experimental group. *Statistically different from synthetic curcumin at the same time by 2-way ANOVA followed Tukey´s test with a $p < 0.05$.

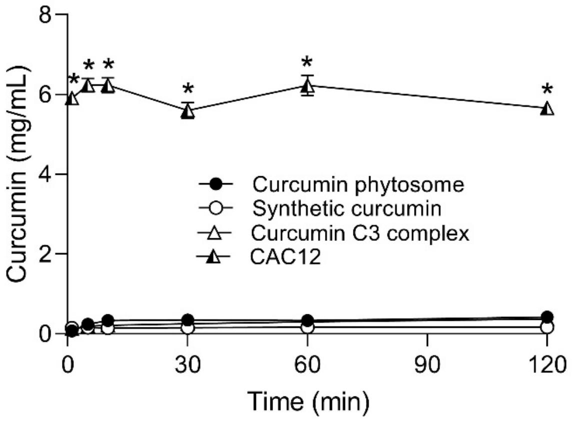

Figure S2. Comparative solubility of curcumin as CAC12 (1:2), curcumin phytosome, curcumin C3 complex or synthetic curcumin in distilled water with 1% of sodium lauryl sulphate. Data are expressed as the mean ± S.E.M. of 3 samples per experimental group. *Statistically different from curcumin phytosome, synthetic curcumin or curcumin C3 complex by 2-way ANOVA followed Tukey´s test with a $p < 0.05$.

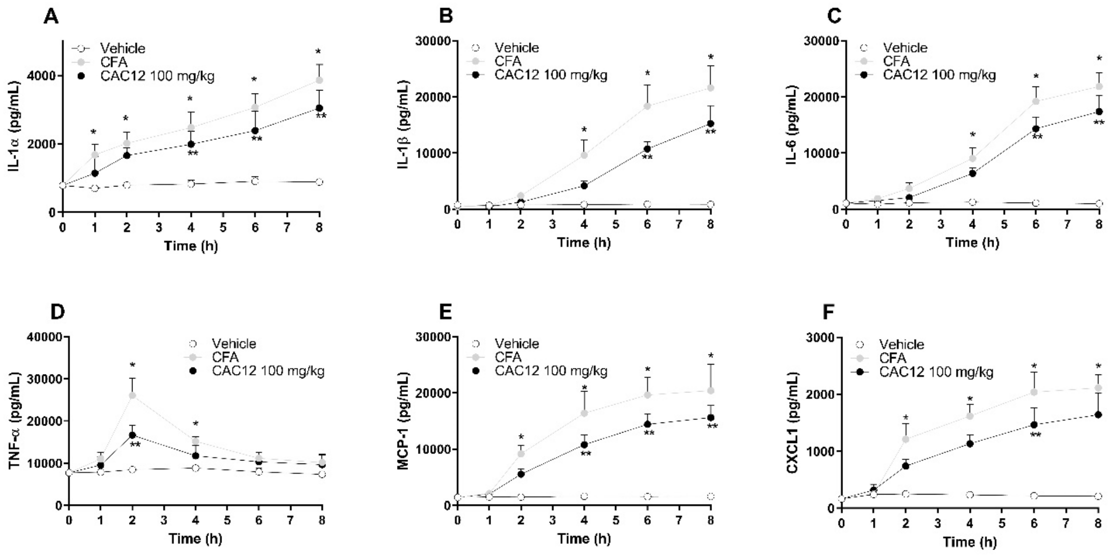

Figure S3. Time courses showing the effect of oral administration of 100 mg/kg CAC12 (1:2) on the levels of interleukin 1 alpha (IL-1α) **(A)**, interleukin 1 beta (IL-1β) **(B)**, interleukin 6 (IL-6) **(C)**, tumor necrosis factor-alpha (TNF-α) **(D)**, monocyte chemoattractant protein 1 (MCP-1) **(E)** and CXC motif chemokine ligand 1 (CXCL1) **(F)**, induced by complete Freund's adjuvant (CFA). Data are expressed as the mean ± S.E.M. of 3-4 animals per experimental group. *Statistically different from the vehicle group and ** statistically different from the CFA group by ANOVA, followed by Tukey´s test with a $p < 0.05$.

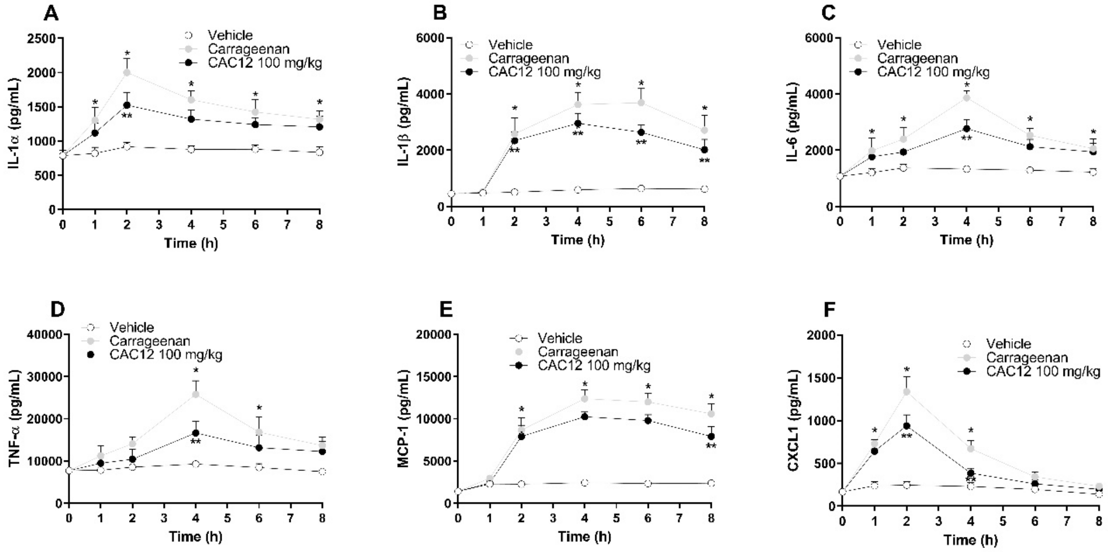

Figure S4. Time courses showing the effect of oral administration of 100 mg/kg CAC12 (1:2) on the levels of interleukin 1 alpha (IL-1α) **(A)**, interleukin 1 beta (IL-1β) **(B)**, interleukin 6 (IL-6) **(C)**, tumor necrosis factor-alpha (TNF-α) **(D)**, monocyte chemoattractant protein 1 (MCP-1) **(E)** and CXC motif chemokine ligand 1 (CXCL1) **(F)**, induced by carrageenan. Data are expressed as the mean ± S.E.M. of 3-4 animals per experimental group. *Statistically different from the vehicle group and ** statistically different from the carrageenan group by ANOVA, followed by Tukey´s test with a $p < 0.05$.

Table S1. Summary of the main vibrational band assignments of curcumin, L-arginine, and the CAC12 by attenuated total reflectance-Fourier transform infrared.

Phase / Vibration	Curcumin (cm^{-1})	L-arginine (cm^{-1})	CAC12 (cm^{-1})
v (O-H) phenol stretching in curcumin	3504	-------	3330
v ($-NH_2$) asymmetric stretching amine in L-arginine	-------	3357	3330[a]
v (N-H) stretching guanidinium in L-arginine	-------	3296	3330[a]
v (O-H) enol stretching in curcumin	3298	-------	3148[b]
v ($-NH_2$) symmetric stretching amine in L-arginine	-------	3041	3148[b]
v (C=O) stretching asymmetric carboxylate group in L-arginine	-------	1718	1666[a]
v (C=N) stretching guanidinium group in L-arginine	-------	1673	1660[a]
v (C=C) enol and (C=O) stretching carbonyl in curcumin	1626	-------	1621
v ($-NH_2$) asymmetric deformation in L-arginine	-------	1613	1621
v (C=O) stretching carbonyl group in curcumin	1496	-------	1510
v (C-OH) in plane deformation phenol in curcumin	1427	-------	1417[a]
v (COO-) asymmetric stretching carboxylate in L-arginine	-------	1418	1417[a]
v (COO-) symmetric stretching carboxylate in L-arginine	-------	1330	1276
v (C-O) enol stretching in curcumin	1268	-------	1276
v (C-O) enol stretching in curcumin	1202	-------	1218
